# European Non-Communicable Respiratory Disease Research, 2002-13: Bibliometric Study of Outputs and Funding

**DOI:** 10.1371/journal.pone.0154197

**Published:** 2016-04-25

**Authors:** Mursheda Begum, Grant Lewison, John S. F. Wright, Elena Pallari, Richard Sullivan

**Affiliations:** 1 Department of Cancer Studies, Division of Cancer Epidemiology and Population Health, Guy's Hospital, King's College London, London, United Kingdom; 2 LSE Health, Department of Social Policy, London School of Economics and Political Science, London, United Kingdom; Charles University in Prague, CZECH REPUBLIC

## Abstract

This study was conducted in order to map European research in chronic respiratory diseases (CRDs). It was intended to assist the European Commission and other research funders to identify gaps and overlaps in their portfolios, and to suggest ways in which they could improve the effectiveness of their support and increase the impact of the research on patient care and on the reduction of the incidence of the CRDs. Articles and reviews were identified in the Web of Science on research in six non-communicable respiratory diseases that were published in 2002–13 from 31 European countries. They represented only 0.8% of biomedical research output but these diseases accounted for 4.7% of the European disease burden, as measured by Disability-Adjusted Life Years (DALYs), so the sub-field is seriously under-researched. Europe is prominent in the sub-field and published 56% of the world total, with the UK the most productive and publishing more than France and Italy, the next two countries, combined. Asthma and Chronic Obstructive Pulmonary Disease (COPD) were the diseases with the most publications and the highest citation rates. They also received the most funding, with around two acknowledgments per paper (in 2009–13), whereas cystic fibrosis and emphysema averaged only one. Just over 37% of papers had no specific funding and depended on institutional support from universities and hospitals.

## Introduction

Chronic Respiratory Diseases (CRDs) include a wide range of non-communicable conditions—such as Chronic Obstructive Pulmonary Disease (COPD), emphysema, allergic rhinitis, asthma, pulmonary arterial hypertension and cystic fibrosis with pulmonary manifestations—which have the common characteristic of adversely affecting patient airways and lung structures [1]. They are usually distinct from communicable ones, such as tuberculosis and influenza.

Across both the developed and the developing world, CRDs are a major public health problem. In developing countries, they are associated with poverty, pollution and poor access to health care resources [2]. In industrialized countries of the European Union, they are associated with increased tobacco-use, obesity, socio-economic inequalities and limited access to healthcare resources [3]. In the year 2000, COPD was the fourth leading cause of mortality responsible for 2.8 million deaths [4]. The World Bank/World Health Organization has projected that by 2020, COPD will rank as the fifth most debilitating condition in the world, in terms of worldwide burden of disease [5].

The two major components of CRDs are COPD and asthma. COPD includes a number of specific lung conditions, characterised by progressive airflow limitation, often a pulmonary response to noxious particles or gases, such as tobacco smoke [6]. It is both progressive and irreversible, and can exacerbate co-morbidities such as lung cancer and cardiovascular disease [7]. COPD is recognized as a systemic inflammatory disorder with numerous additional pulmonary and extrapulmonary manifestations, including an increased risk for development of primary lung cancers [8]. It can also lead to weight loss and skeletal muscle dysfunction [9]. Asthma is caused by the hyper-responsiveness of the bronchi and trachea to particular stimuli and occurs through narrowing of the airways; it appears to be occasioned by both environmental and genetic factors [10]. Symptoms include tightness in the chest, coughing, wheezing and shortness of breath, and can be alleviated by means of inhalers with bronchodilators or glucocorticosteriods [11].

World-wide, the prevalence of these CRDs is increasing and the percentage of deaths rose from 6.5% in 2002 to 7.2% in 2010, although many sufferers, probably the majority, are not actually diagnosed. Within Europe, the severity of the burden varies greatly, being over 7% in the UK in 2010 in terms of Disability-Adjusted Life Years (DALYs), but less than 2% in Estonia. In most European countries, the relative burden has decreased since 2002, according to the WHO Global Burden of Disease estimates from the University of Washington's Institute for Health Metrics and Evaluation (IHME: http://vizhub.healthdata.org/gbd-compare/), and the European average has gone down from 5.4% of all DALYs to 4.5%. This probably reflects the reductions in smoking and coal-mining, and improvements in air quality. However, in the 11 former socialist countries of central and eastern Europe that are now EU Member States, the burden has gone up from 3.06% to 3.36% and it has increased markedly in Bulgaria and Poland. The two main diseases are COPD and asthma, averaging 2.9% of all DALYs and 1.1% respectively in Europe in 2010; the others, including cystic fibrosis, only accounted for 0.7% of DALYs.

The recent reductions in smoking in western European countries (*e*.*g*., from 28% to 22% in England between 1998 and 2008 [12], and much more in Scandinavia, [13]) will certainly have helped to reduce the disease burden, especially for cancer, cardiovascular disease and diabetes, but also for these chronic respiratory diseases. There have also been parallel bans on smoking in enclosed spaces in the USA, and they have led to declines in acute myocardial infarction [14, 15]. The effects of smoking on asthma and COPD have been described in some detail [16, 17]; they are exacerbated by industrial pollutants [18, 19]. In eastern Europe, and many other countries, smoking is still increasing, particularly among women, with consequent effects on respiratory diseases. The effects of global warming on climate change may also affect the incidence of asthma [20, 21], allergic rhinitis (often caused by pollen from ragweed [22, 23]) and COPD [24].

There appear to be few papers on the outputs of research on these diseases although there are two on environmental tobacco smoke research [25, 26]. One study revealed that only a minority of abstracts of clinical trials of treatments for CF presented at conferences were subsequently published in the serial literature [27]. A study on the Spanish-language journal, *Archivos de Bronconeumologia*, from 1970 to 2000 with particular reference to research on smoking, looked at the bibliometric characteristics of the articles [28] but did not find any striking results. A more comprehensive study of all respiratory disease research, including that on lung cancer, showed that Finland, Canada, Spain and the UK had the greatest relative commitment to respiratory disease research expressed as a ratio of their share of world biomedical research, and that charitable funding helped the UK to score well in CF research [29]. Recently, there has been a study on respiratory research outputs from China, Hong Kong and Taiwan; the former has now overtaken both of the others in output but its research remains less well cited [30].

The research described here was carried out as part of an investigation for the European Union (EU) on the outputs of research on five non-communicable diseases (NCDs) in Europe. This was intended to reveal gaps and overlaps, and to learn more about the funding sources for this research. [The other four NCDs are cancer, cardiovascular disease (including stroke), diabetes and mental disorders, and the findings of the project on these will be published separately.] Funding for research on CRDs, as for other medical conditions, comes from four main sources: national and regional government, private-non-profit organisations (including collecting charities, endowed foundations, hospitals and universities, and voluntary non-profit associations), industry and international bodies such as the EU. This project was intended to assist them to understand the research environment in Europe, and to suggest ways in which they could be more effective in their support of research and of activities that would promote its transfer to the care of patients and public health campaigns to prevent illness through changes in behaviour. It was also intended to inform the researchers on the areas of research in need of more attention, and of which countries could provide relevant expertise to those ones wishing to improve their capability. The researchers could also use the findings to seek more funding from external funders and from internal sources.

## Methodology

The bibliographic details of papers recorded in the Web of Science published by Thomson Reuters (WoS) were identified by means of a special filter. This was based on title words for the names of the respiratory diseases separated with Boolean operator “OR” (“*asthma OR bronchiectasis OR cystic fibrosis OR CFTR OR COPD OR chronic obstructive respiratory disease OR emphysema OR mucoviscidosis”*), and four specialist journals: “*COPD-Journal of Chronic Obstructive Pulmonary Disease* OR *International Journal of Chronic Obstructive Pulmonary Disease* OR *Journal of Asthma* OR *Journal of Cystic Fibrosis”*. Papers were selected if they had one of the title words, or were in one of the specialist journals, or both. The WoS software combines the results of separate search statements so that duplicates are eliminated.

"Articles" and "reviews" (as defined in the WoS) from the 12 years, 2002–13, with an address in one or more of the 28 European Union Member States, plus Iceland, Norway and Switzerland, were downloaded to a series of files, 500 at a time. This time frame allowed an adequate time period for any recent trends to be observed. The list of countries is shown in Table 1, with their digraph International Standards Organisation (ISO2) codes. They were then transferred to an Excel spreadsheet by means of a macro written by Philip Roe of Evaluametrics Ltd, St Albans, UK. The addresses were parsed to show the fractional count of each country in each paper. (A paper with one German and two French addresses would be counted 0.33 for Germany and 0.67 for France.) Five-year citation counts were determined for the papers published in 2002–09 to allow fair comparisons between later and earlier papers since previous research in the domain by the authors have used the same fixed window of time.

**Table 1:** List of the 31 European countries whose outputs were examined in this study with their ISO2 codes.

ISO	Country	ISO	Country	ISO	Country	ISO	Country
**AT**	Austria	**EE**	Estonia	**IS**	Iceland	**PL**	Poland
**BE**	Belgium	**ES**	Spain	**IT**	Italy	**PT**	Portugal
**BG**	Bulgaria	**FI**	Finland	**LT**	Lithuania	**RO**	Romania
**CH**	Switzerland	**FR**	France	**LU**	Luxembourg	**SE**	Sweden
**CY**	Cyprus	**GR**	Greece	**LV**	Latvia	**SI**	Slovenia
**CZ**	Czech Rep.	**HR**	Croatia	**MT**	Malta	**SK**	Slovakia
**DE**	Germany	**HU**	Hungary	**NL**	Netherlands	**UK**	United Kingdom
**DK**	Denmark	**IE**	Ireland	**NO**	Norway		

However, the impact of medical research can be better gauged from its influence on clinical practice. This is difficult to judge, but a proxy indicator is the extent to which it has formed the evidence base of clinical guidelines, which are being developed and published in increasing numbers. As part of the EU mapping project, we collected these from 19 different countries, numbering 45 in total, and matched their cited references to papers in the WoS. We thereby compiled a database of 7184 references, and could compare the numbers from each European country, and their percentage of the European total, with the presence of each country in respiratory disease research. This was done on a fractional count basis, both for asthma and for COPD. Some papers would have been cited on many different guidelines; this would indicate their importance for guiding clinical practice in Europe.

Comparisons were made between the outputs of the 31 European countries in respiratory disease research (RESPI) and their wealth, as measured by their Gross Domestic Products (GDP). Several previous studies [31,32] have demonstrated that there is a close correlation between these two indicators; they are normally plotted on log-log scales because of their wide variation in values (several orders of magnitude). The best (least-squares) regression line to fit the data is based on a power-law, and appears as a straight line on log-log paper. Departures from this line can then show which countries are performing particularly well or badly, with observed outputs being compared with those expected on a Poisson distribution with one degree of freedom.

Country relative commitments (RC) to RESPI research were compared with their biomedical research output in the same years. This was based on a special address filter that was originally developed to distinguish between biomedical and other papers in multi-disciplinary journals such as *Nature* and *Science*. These were based on integer counts. For research on the individual diseases, comparisons were made with the European average, and were based on fractional counts.

Details of the funding sources were obtained from the WoS for papers from 2009–13. The list of funders given in the WoS included many false positives where companies had remunerated authors for unrelated work declared in a statement of Conflict of Interest [33]. These statements were first identified by means of another macro, and then individually read to redact the list of explicit funders. There were also implicit funders taken from the addresses–government research laboratories, charity laboratories, and commercial companies. Papers without either type of funding would have received institutional support from a university or a hospital.

Because the names of the funders were not standardised, it was necessary to give them codes so that they could be individually identified. These were in three parts: a trigraph to identify the funder, *e*.*g*., MRC = UK Medical Research Council; a digraph to show their sector and sub-sector (see Table 2); and their ISO digraph code. The thesaurus contained almost 12,000 individual funders, but small funding sources, of which there were very many, were given "generic" codes that simply identified their sub-sector and country. All codes were manually applied with the aid of two thesauruses which were developed from previous work; one of funders and the other of author addresses.

**Table 2:** List of sectors and sub-sectors used for the classification of research funders. *GOV = government; PNP = private-non-profit; INDY = industry*.

Sector	Code	Sub-sector	Sector	Code	Sub-sector
**GOV**	GA	Government agency	**PNP**	CH	Collecting charity
	GD	Government department		FO	Endowed foundation
	LA	Local authority		HT	Hospital trustees
				MI	Mixed (academic)
**INDY**	BT	Biotechnology company		NP	Other non-profit
	IN	Industrial (non-pharma)			
	IP	Pharmaceutical			
	SN	Subsidiary of industry			
	SP	Subsidiary of pharma			

Finally, we carried out a survey of some leading researchers in several European countries with different levels of income *per caput* in order to try and determine the average cost of a published paper in the five NCDs. We asked the researchers for their total annual research budgets during the last five years (2009–13) and compared their responses with their outputs of papers, fractionated according to the numbers of addresses. These results were then compared with results of earlier surveys carried out with the same methodology [34, 35] that had been used to determine the cost of a biomedical research paper in other subject areas.

### Disease burden

In order to put our research results in context, we sought data on the burden from these non-communicable respiratory diseases in Europe. Data on the 31 European countries were downloaded for the year 2010 from the IMHE website and revealed that these diseases accounted, on average, for 4.7% of all Disability-Adjusted Life Years (DALYs), including 2.9% for COPD and 1.1% for asthma.

**Fig 1:**
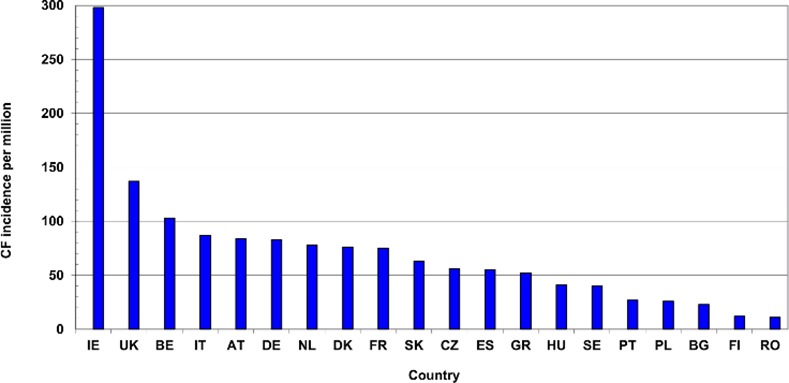
Incidence of cystic fibrosis in selected European countries [36].

The countries suffering most from asthma are the UK (1.8% of DALYs), Ireland (1.7%), Portugal, Cyprus and Sweden (1.6%). But the "accession" countries in eastern Europe suffer much less, with the lowest burdens being in Lithuania, Bulgaria and Latvia (about 0.4%). In COPD the relative burden is greatest in Denmark (4.7%), Switzerland (4.5%) and the UK (4.2%), and least in the Baltic countries: Estonia (1.2%), Latvia (1.4%), and Lithuania and Finland (2.0%). Burden of disease data are not given by the IHME for cystic fibrosis, but surveys in European countries have been made of the incidence of the disease and reported by Farell [36]. The results for the EU27 Member States are shown in Fig 1 except for the seven countries with populations below 4 million. They can be compared with the results of surveys of the genetic mutation (DF508) that is mainly responsible for the disease [37]. Both surveys put Ireland in first place; the UK and Italy also have a high incidence of CF. However, it is much lower in eastern Europe (Poland, Bulgaria and Romania) and in the Baltic countries (data for Latvia and Lithuania not shown in Fig 1, but below 14 per million), although Estonia is an exception

## Results

### Respiratory research outputs and relative commitments

**Fig 2:**
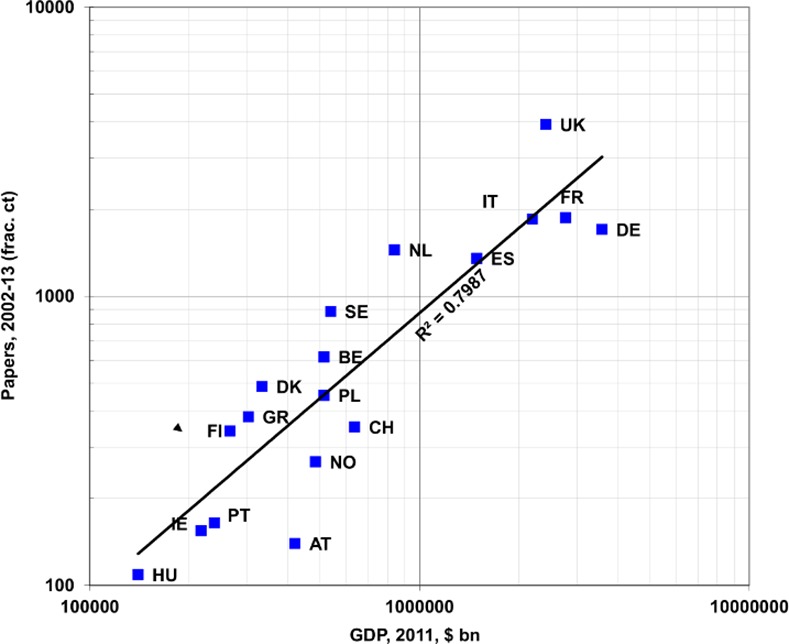
Plot of RESPI paper output, 2002–13, against GDP for 18 European countries with fractional counts above 100 papers. Note: BG, CY, CZ, EE, HR, IS, LT, LU, LV, MT, RO, SI and SK omitted. For codes, see Table 1.

In the 12 years, 2002–13, there were 18,822 European RESPI papers in the WoS, representing 56% of the world total of 33,629. The European total was only 0.8% of their biomedical output of 2,442,063 papers in 2002–13. The European output increased by 75%, and the international fraction (*i*.*e*., the difference between the integer total for the 31 countries and the fractional total, divided by the former) doubled from 6.2% to 12.4%. These percentages are similar to those for the other NCDs. Fig 2 shows the outputs of the 18 leading countries on a fractional count basis and a comparison with the countries' wealth. The regression-line that best fits the data on a least-squares basis is a power-law one with number of papers = 0.0012 * GDP ^0.9753^, a straight line on a log-log plot. The association is high (r^2^ = 0.80), but some countries publish about twice what the regression line would suggest, such as the Netherlands (1447 compared with 720), Sweden (886 compared with 446), and the UK (3924 compared with 2018), and others such as Austria (140 compared with 365) less than half. These departures from the expected numbers of papers are statistically highly significant (p << 0.01%), based on a Poisson distribution with one degree of freedom.

The outputs of the leading European countries in RESPI compared with their presence in biomedical research, and the percentage of their DALYs attributable to non-communicable respiratory diseases in 2010, are shown in Table 3 on an integer count basis. Values more than sqrt(2), less than sqrt (0.5) or less than 0.5 are shown in a different font. All of the differences between observed values and those expected on the basis of the European average are very highly significant on the Poisson distribution with one d/f except for ES, GR, and PT where p < 0.1%; for PL and IE where p < 5%, and FR, IT, FI and NO which are not statistically significant.

**Table 3:** Percentage of their DALYs attributable to RESPI diseases in 2010 of 18 leading European countries and their percentage presence (integer counts) in non-communicable respiratory disease research and in all biomedical research, 2002–13.

	% DALYs	RESPI papers	% of world	Biomed papers	% of world	RC to RESPI	RESPI/biomed, %
**UK**	7.1	5537	16.5	568306	9.3	**1.77**	0.97
**DE**	4.5	2474	7.4	491252	8.0	0.91	*0*.*50*
**FR**	4.2	2387	7.1	311583	5.1	1.39	0.77
**IT**	4.2	2372	7.1	294964	4.8	**1.46**	0.80
**NL**	5.3	2065	6.1	203963	3.3	**1.84**	1.01
**ES**	4.8	1742	5.2	200964	3.3	**1.57**	0.87
**SE**	5.0	1407	4.2	134251	2.2	**1.90**	1.05
**BE**	5.6	990	2.9	103663	1.7	**1.73**	0.96
**DK**	6.4	792	2.4	79304	1.3	**1.81**	1.00
**CH**	6.6	695	2.1	133055	2.2	0.95	*0*.*52*
**PL**	4.0	580	1.7	80694	1.3	1.31	0.72
**GR**	5.6	510	1.5	54017	0.9	**1.72**	0.94
**FI**	3.5	489	1.5	59216	1.0	**1.50**	0.83
**NO**	5.0	458	1.4	52619	0.9	**1.58**	0.87
**AT**	4.8	263	0.8	70010	1.1	*0*.*68*	*0*.*38*
**IE**	5.8	239	0.7	35507	0.6	1.22	*0*.*67*
**PT**	5.1	225	0.7	36704	0.6	1.11	*0*.*61*
**HU**	4.2	158	0.5	31979	0.5	0.90	*0*.*49*

The ratio = their relative commitment (RC) to RESPI. RC values and % of biomed > 1.41 in **bold**, < 0.71 in *italics*, < 0.5 in *small italics*. *Codes are given in Table 1.*

Ten of these 18 countries have a relative commitment to respiratory research greater than 1.41: this means that there are many non-European countries with a much lower RC to the subject area. But only two have their output in respiratory disease research above 1% of biomedical research (Sweden and the Netherlands) and their disease burden is 5% or higher, as is that in eight other countries. On average, the percentage of biomedical research in RESPI is only 15% of the percentage of the disease burden in DALYs.

The European countries varied in their concentration on the different respiratory diseases. Data are given in Table 4 only for the three main ones: asthma (AST) with 7563 papers (40% of the total of 18,822 RESPI papers), COPD (COP) with 4763 papers (25%) and cystic fibrosis (CYF) with 3281 (17%). The countries that concentrate most on asthma are Finland and Poland, with Ireland doing relatively little. In COPD, the leaders are Spain, Greece, the Netherlands and Norway in terms of relative commitment, and in cystic fibrosis Ireland stands out, which is appropriate, given its very high incidence of the disease (see Fig 2), together with Portugal and France. Finland does very little CF research, which also accords with its low incidence of the disease.

**Table 4:** Numbers of RESPI papers in the three main non-communicable respiratory diseases from each of the 18 leading European countries, 2002–13, fractional counts, and their relative commitment (RC) to research on each disease relative to their output in RESPI and to the European average.

	**Number of papers**				**Relative commitment**		
ISO2	*AST*	*COP*	*CYF*	*RESPI*	*AST*	*COP*	*CYF*
**UK**	1747	1068	921	3924	1.11	1.08	1.35
**FR**	848	320	555	1870	1.13	*0*.*68*	**1.70**
**IT**	736	560	361	1847	(0.99)	1.20	1.12
**DE**	686	364	415	1701	(1.00)	0.85	1.40
**NL**	643	591	163	1447	1.11	**1.61**	*0*.*65*
**ES**	504	585	143	1351	0.93	**1.71**	*0*.*61*
**SE**	503	217	77.8	886	1.41	(0.97)	*0*.*50*
**BE**	218	158	163	617	0.88	(1.01)	**1.52**
**DK**	227	154	90.5	487	1.16	1.25	(1.07)
**PL**	295	78.3	56.3	454	**1.62**	*0*.*68*	0.71
**GR**	167	166	35.6	383	(1.09)	**1.71**	*0*.*53*
**CH**	140	96.2	80.4	353	(0.99)	(1.08)	1.31
**FI**	262	58	4.25	342	**1.91**	*0*.*67*	*0*.*07*
**NO**	138	109	14.6	267	1.29	**1.61**	*0*.*31*
**PT**	77.9	28	50.9	164	1.18	*0*.*67*	**1.78**
**IE**	29.7	24.5	90.1	155	*0*.*48*	*0*.*62*	**3.33**
**AT**	63.6	38.5	21.2	140	(1.13)	(1.09)	(0.87)
**HU**	65	16	10.4	109	**1.48**	*0*.*58*	*0*.*55*
**Total**	7563	4763	3281	18822			

RC values > 2.0 in **large bold**, > 1.41 in **bold**, < 0.71 in *italics*, < 0.5 in *small italics*. Values with statistical significance p < 0.001% shown underscored; those not statistically different from unity shown in (parentheses), based on the Poisson distribution with one d/f. *Country codes are given in Table 1.*

### Citation scores

European RESPI papers were slightly more highly cited than the world average. The number of citations received in the five years following publication (Actual Citation Impact, ACI) increased from 14.9 (+ 0.48) in 2002–03 to 16.2 (+ 0.56) in 2008–09 (figures in parentheses are the standard errors of the mean, s.e.m.). These compare with world average values of 13.8 and 14.3. Within Europe, the best-cited papers were those from the UK, see Table 5, which gives the mean ACI values and the percentages of the countries' papers with enough five-year citations (52) to put them in the top 5% of European papers, both on a fractional count basis. On this basis, it is not possible to give values for the s.e.m. as the citation counts are each multiplied by the fractional presence of each country in the paper.

**Table 5:** Citation performance of 18 EUR31 countries in RESPI in 2002–09 with at least 50 citable papers, ranked by the percent with 52 or more cites in the five years following publication (ACI) (Top 5%) rather than the mean value, fractional counts.

ISO	ACI	Top 5%	%	ISO	ACI	Top 5%	%	ISO	ACI	Top 5%	%
**UK**	19.6	176.4	7.38	**NO**	14.7	5.4	3.80	**FI**	14.1	5.6	2.44
**BE**	18.2	24.8	6.74	**ES**	12.2	23.7	3.12	**GR**	10.1	3.6	1.75
**DK**	18.3	15.3	6.04	**IT**	12.9	35.1	3.18	**HU**	11.3	1.0	1.75
**NL**	17.9	45.1	5.32	**IE**	11.9	2.0	3.0	**PL**	8.5	3.9	1.69
**CH**	16.0	9.6	4.64	**FR**	9.8	34.9	2.80	**AT**	11.4	1.2	1.37
**DE**	13.9	43.9	4.04	**SE**	13.7	13.8	2.55	**PT**	8.3	0.3	0.33

A country's presence in the top 5% of papers is a rather more sensitive measure of impact than mean citation score (ACI) as these are the important papers that are likely to influence research and treatment.

Papers on COPD were more highly cited than those in asthma and cystic fibrosis, see Table 6.

**Table 6:** Five-year citation scores, and numbers of papers in the top 5% (52 cites), top 1% (121 cites) and top 0.2% (250 cites) in three respiratory disease areas, 2002–09.

Disease	Cit.	ACI	52 c	Top 5%	121 c	Top 1%	250 c	Top 0.2%
**Asthma**	5444	16.7 ±0.41	304	5.58	65	1.19	13	0.24
**CF**	2168	11.7 ±0.33	51	2.35	5	0.23	0	0.00
**COPD**	2728	19.5 ±0.79	202	7.40	43	1.58	9	0.33
**RESPI**	11207	16.0 ±0.29	573	5.11	113	1.01	21	0.19

### Citations on clinical guidelines

Of the 7184 references, 3744 were to the 23 clinical guidelines on COPD and 3052 were to the 20 on asthma. Table 7 shows the numbers of references (on a fractional count basis) from the 16 leading countries in each disease area, and for comparison, the numbers expected from the countries' fractional count presence in COPD and asthma research, see Table 4.

**Table 7:** Citations of European RESPI papers on 45 European respiratory clinical guidelines (CGs), and comparison with research outputs (Res): percentages of EUR31 total.

	*Asthma*				*COPD*			
*Country*	*Res*, *%*	*CGs*	*CGs*, *%*	*Ratio*	*Res*, *%*	*CGs*	*CGs*, *%*	*Ratio*
UK	23.1	608	40.5	**1.75**	22.4	683	36.1	**1.61**
NL	8.5	145	9.6	1.13	12.4	259	13.7	1.10
IT	9.7	119	7.9	0.81	11.8	152	8.0	*0*.*68*
ES	6.7	59.5	4.0	*0*.*59*	12.3	186	9.8	0.80
SE	6.7	105	7.0	1.05	4.6	85.4	4.5	0.99
DE	9.1	85.3	5.7	*0*.*63*	7.6	81.6	4.3	*0*.*56*
DK	3.0	86.1	5.7	**1.91**	3.2	86.4	4.6	**1.41**
FR	11.2	70.1	4.7	*0*.*42*	6.7	82.6	4.4	*0*.*65*
BE	2.9	45.3	3.0	1.05	3.3	86.1	4.5	1.37
FI	3.5	78.3	5.2	**1.50**	1.2	26.1	1.4	1.13
CH	1.9	17.4	1.2	*0*.*62*	2.0	61.4	3.2	**1.61**
NO	1.8	24.1	1.6	0.88	2.3	36.8	1.9	0.85
PL	3.9	13.5	0.9	*0*.*23*	1.6	19.3	1.0	*0*.*62*
GR	2.2	6.2	0.4	*0*.*19*	3.5	15.4	0.8	*0*.*23*
IE	0.4	5.5	0.4	0.92	0.5	14.3	0.8	**1.47**
AT	0.8	10.4	0.7	0.83	0.8	5.4	0.3	*0*.*35*

Ratios > 1.41 in **bold**, ratios < 0.71 in *italics*, < 0.5 in *small italics*.

The countries whose research contributes most to the evidence base of European clinical guidelines, relative to its volume, are the UK and Denmark for both asthma and COPD. In asthma Finland's research is well-cited and in COPD this is so for Switzerland and Ireland. On the other hand, research from Italy, Spain, Germany, Austria, France, Poland and Greece has relatively less influence.

### Funding of respiratory disease research

**Fig 3:**
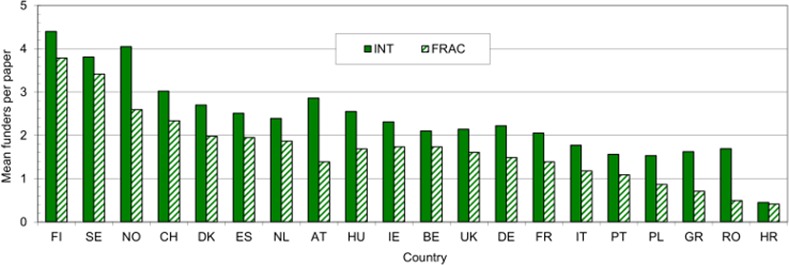
Mean numbers of funding acknowledgments per paper, explicit and implicit, on the basis of integer paper counts (INT) and of fractional counts (FRAC) for the 20 leading European producers of RESPI papers, 2009–13.

The analysis of funding, both explicit acknowledgments and ones implicit from the addresses, shows that RESPI research does not attract much specific support, and over 37% of 2009–13 papers do not have it. The mean number of funders per paper overall was 1.98, and one paper had over 100, but there was considerable variation between countries, see Fig 3.

The countries whose papers obtained the most funding, often given to their partners in international collaborations, were three Scandinavian ones (Finland, Sweden and Norway), and Denmark was in fifth place in this presentation. [Iceland would have been easily first, but published only 16 papers in 2009–13 on a fractional count basis.] Central and Eastern European countries attracted little funding except from their own governments, though Hungary was an exception, probably because of extensive international collaboration. Croatia (HR), in southeast Europe, was relatively scientifically isolated, and 30 of its 49 papers acknowledged no specific funding source.

**Fig 4:**
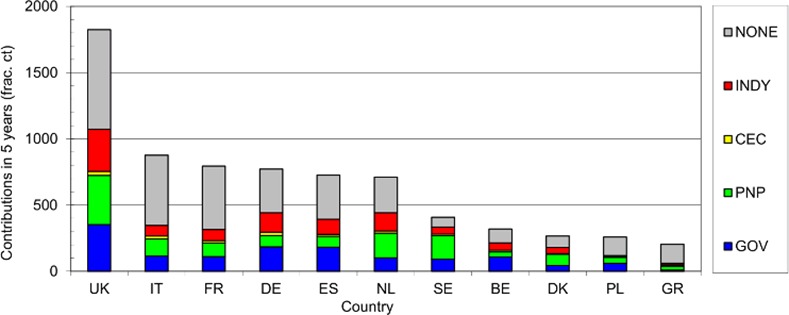
Main sectors supporting respiratory disease research in 11 leading European countries, 2009–13. GOV = public sector (including local authorities), PNP = private-non-profit, CEC = European Union, INDY = industry (mainly pharma).

An analysis was also conducted of the leading funders in terms of fractional credit, both overall and for the leading countries, and of their sectors (government, private-non-profit, industrial and international). The results for the leading funders are in Table 8, and the chart showing how the leading countries' support is provided is in Fig 4. Estimates of annual expenditure are based on our estimates of the mean cost of a paper (see below). However, the expenditure by pharma companies is likely to be far higher as they will be carrying out intramural work that does not lead to open publications.

**Table 8:** List of leading funders of European respiratory disease research, 2009–13, with fractional counts of numbers of papers (N) and estimates of annual research funding for European papers (€ million).

Code	Funders	N	€ M/yr
GSW-IP-UK	GlaxoSmithKline plc	233	12.1
CEC-GD-EU	European Union	223	11.6
DOH-GD-UK	UK Department of Health (including NHS hospitals)	169	8.8
ZAT-IP-UK	AstraZeneca plc	157	8.1
DFG-GA-DE	Deutsche Forschungsgesellschaft	126	6.5
NVP-IP-CH	Novartis s.a.	123	6.4
ESS-GA-ES	Spanish Institute Carlos III	97	5.0
INS-GA-FR	French INSERM	85	4.4
DUA-CH-NL	Netherlands Asthma Foundation (charity)	84	4.4
MRC-GA-UK	UK Medical Research Council	84	4.4
BOI-IP-DE	Boehringer Ingelheim AG	79	4.1
WEL-FO-UK	Wellcome Trust, London	78	4.0
PFZ-IP-US	Pfizer Inc.	68	3.5
MRK-IP-US	Merck Inc.	64	3.3
Y59-NP-ES	Spanish non-profit organisations	51	2.7
FSK-LA-BE	Fonds voor Wetenschappelijk Onderzoek Vlaanderen	50	2.6
SHL-CH-SE	Swedish Heart and Lung Foundation (charity)	47	2.4
FRC-CH-FR	Vaincre la Mucoviscidose (French Cystic Fibrosis F'd'n)	46	2.4
Z08-MI-PL	Polish universities	45	2.4
TAK-IP-JP	Takeda Ltd	42	2.2
AST-CH-UK	Asthma UK (charity)	41	2.1
FCU-CH-IT	Fondazione Italiana per la Fibrosi Cistica (charity)	41	2.1
POM-GD-PL	Polish Ministry of Science and Higher Education	40	2.1
SHD-GD-UK	Scottish government	40	2.1

In this analysis, it has been assumed that national public and private-non-profit funders only support researchers in their own country, that European Union funding goes to the European countries whose addresses are on the paper in proportion to their number, and that industrial funding can go to any country. It is striking that pharmaceutical companies appear so prominently in this list, with six in the top 18.

Support from the European Union (EU) comes from a variety of sources, mostly but not exclusively the Framework Programmes. Table 9 shows the countries that receive the most support (fractional count of papers), and also the ones where the EU support is proportionately the greatest (Latvia, Slovakia and the Czech Republic).

**Table 9:** European Union support for respiratory disease research, 2009–13: numbers of papers (left columns) and percent of papers for individual countries (EU, %, right columns).

*Country*	*Papers*	*EU*, *%*	*Country*	*Papers*	*EU*, *%*	*Country*	*Papers*	*EU*, *%*
**UK**	**31.5**	**1.7**	**CH**	**7.1**	**4.1**	**IE**	**2.8**	**3.0**
DE	27.2	3.5	PL	5.8	2.3	LV	2.0	47.1
IT	22.8	2.6	FI	5.1	3.8	IS	0.7	4.3
NL	17.3	2.4	SK	4.7	20.3	RO	0.5	1.1
FR	17.0	2.1	DK	4.6	1.7	SI	0.4	1.0
ES	16.9	2.3	CZ	4.5	11.4	LT	0.4	2.0
BE	12.4	3.9	NO	4.0	2.9	EE	0.3	5.2
SE	10.8	2.6	HU	3.9	6.9	CY	0.2	2.9
GR	9.2	4.5	AT	3.4	6.1	BG	0.1	0.4
PT	9.0	8.4				**Total**	**224.5**	**2.7**

None of the RESPI papers from Croatia, Luxembourg and Malta were funded by the EU.

This table reveals clearly that the large countries in scientific output received by far the bulk of EU support (the top seven obtained almost two thirds of the total), but some of the smaller countries, notably Latvia, Slovakia and the Czech Republic, depended highly on the EU for their research on RESPI.

Our survey of leading researchers elicited some 30 responses, of which 22 gave useable data (some suggested unrealistically high or low costs for a paper). As expected, the responses from high-income countries (Norway and Switzerland) gave a figure (€412 k) higher than that from the researchers in middle-income countries (Belgium and Finland; €260 k), and much higher than that from respondents in low-income countries (Bulgaria, Czech Republic, Romania, Slovakia and Slovenia; €142 k). These mean costs parallel the differences in income *per caput* between the groups, but are less divergent. Since the large majority (about 85%) of all RESPI papers came from middle-income countries (mainly France, Germany, Italy and the UK), the overall average cost per paper worked out at close to their figure, at €255k. (This was also similar to results found in earlier surveys, adjusted for inflation [34,35].) This means that the average annual research expenditure on respiratory diseases in Europe during these years was of the order of €407 million, barely one fifteenth of that on cancer research (€5977 million p.a.). Since the burden from respiratory diseases in Europe is just over one quarter of that from cancer, it is apparent that respiratory disease research is relatively under-funded. If it were to receive as much research money as cancer in relation to its disease burden, it would need an additional €1225 million per year, or three times its existing research budget.

## Discussion

This study has mapped the main features of research into non-communicable respiratory diseases in Europe in 2002–13, the outputs of the 31 countries and their citation scores, and the funding sources for papers in the last five years (2009–13). The study has some limitations. We were not able to check how comprehensive the filter was at capturing relevant papers, and indeed we expect that it will have omitted some basic research papers where the title or the journal did not directly indicate the name of the disease. However, this limitation affects other disease-related research topics. The restriction of the database to articles and reviews is standard bibliometric practice, although it is arguable that other document types such as editorials, letters and meeting abstracts sometimes contain interesting results, and are indeed occasionally cited among the references on clinical guidelines.

The large number of funding bodies acknowledged on the Scandinavian papers is attributable to the many small (and some large) endowed foundations in these countries. In Finland, private-non-profit sources accounted for over half the total funding (54%), and most of these were endowed foundations. (So far, we have encountered over 50 different named Finnish foundations, although not all of them support respiratory disease research.) In the former socialist countries of central and eastern Europe, although there are some private-non-profit funders, notably professional associations, there does not appear to be a tradition of public charitable funding of medical research, or the creation of foundations by wealthy families and individuals. The fiscal regime may also not be so favourable to such activities as it is in western Europe (and north America).

Of the main respiratory diseases, asthma is better established as a research subject than COPD; its integer count output from the EUR31 countries was 8489 papers in 2002–13, or 0.35% of all biomedical research output. This is still much lower than its disease burden of 1.07%, but the ratio is more favourable than for COPD which had 5286 papers or 0.22% compared with a burden of 2.95%, an adverse ratio of 13.6 compared with 3.1 for asthma.

Why should this be? "COPD mainly affects people over the age of 40 and becomes more common with increasing age. The average age when it is formally diagnosed is around 67 years. It is more common in men than women. COPD accounts for more time off work than any other illness and a flare-up (exacerbation) of COPD is one of the most common reasons people seek emergency hospital assistance" [38]. On the other hand, asthma affects people of all ages, and in England one in five sufferers are children, over 1.1 million, or one in 11 children. It is not surprising, therefore, that asthma has a far higher public profile, and there are medical research charities to support asthma research whereas COPD has largely to rely on public funding, plus support from pharma companies. Moreover, because it is so often caused by smoking, it does not attract public sympathy in the same way as asthma.

There are also charities in at least eight European countries (Belgium, France, Germany, Ireland, Italy, Netherlands, Sweden and the UK) that support research in cystic fibrosis, sometimes called mucoviscidosis. Inevitably this disease mainly affects young people as life expectancy for patients is quite low, though it has increased to about 40 years [39] as better treatments have become available.

It appears, therefore, that COPD has an image problem, and does not attract research funding from the European public, who are understandably more concerned with the health of children than with that of old men, many of whom have been smokers. However, COPD is an unpleasant disease, and as the quotation above suggests, it is an expensive one to treat because of the numbers of patients and the difficulty of doing more than provide palliative care. It should therefore have a greater claim on public sources of research funding. There should not be a problem in finding applications of high quality, as COPD research is actually better cited than papers in asthma or CF, as Table 6 shows.

We have not analysed the outputs by the type of research, such as epidemiology, genetics, pharmaceutical and other treatments, and palliative care. This would provide information on which aspects of research may be neglected relative to others and might suggest changes in approach in individual countries. In particular, this would show the types of research that are most frequently cited on clinical guidelines, and so are of medical utility.

## Conclusions

The main conclusion is that respiratory disease research is seriously under-funded. The research outputs of the leading European countries vary between 0.4% of all their biomedical research (Austria) and just over 1.0% (Netherlands, Sweden and Denmark), but these values are all very small compared with the European disease burden of 4.7% of all DALYs. If respiratory disease research were to be funded on the same scale as cancer, relative to its disease burden, funding would need to be **quadrupled**. And within RESPI research, the main need is for more work on COPD, which is even more under-resourced than asthma research but causes more distress and expense.

## References

[1] Bousquet J, Dahl R, Khaltaev N. Global Alliance against chronic respiratory diseases. Eur Respir J. 2007; 29(2): 233–239. 17264322 10.1183/09031936.00138606

[2] Aıt-Khaled N, Enarson D, Bousquet J. Chronic respiratory diseases in developing countries: the burden and strategies for prevention and management. Bull World Health Organ. 2001; 79(10): 971–979. 11693980 PMC2566677

[3] Yach D, Hawkes C, Gould CL, Hofman KJ. The global burden of chronic diseases: overcoming impediments to prevention and control. JAMA. 2004; 291 (21): 2616–2622. 15173153 10.1001/jama.291.21.2616

[4] Mannino DM, Kiri VA. Changing the burden of COPD mortality. Int J Chron Obstr Pulm Dis 2006; 1(3): 219–2335. 10.2147/copd.2006.1.3.219 PMC2707151 18046859

[5] World Bank. *Human development and Public services*: *About the Program* Available: http://econ.worldbank.org/WBSITE/EXTERNAL/EXTDEC/EXTRESEARCH/EXTPROGRAMS/EXTPUBSERV/0,contentMDK:20292625~menuPK:477923~pagePK:64168182~piPK:64168060~theSitePK:477916,00.html. Accessed 10 February 2015.

[6] Trupin L, Earnest G, San Pedro M, Balmes JR, Eisner MD, Yelin E, et al The occupational burden of chronic obstructive pulmonary disease. Eur Respir J. 2003; 22(3): 462–469. 14516136 10.1183/09031936.03.00094203

[7] Qaseem A, Wilt TJ, Weinberger SE, Hanania NA, Criner G, van der Molen T, et al Diagnosis and management of stable chronic obstructive pulmonary disease: a clinical practice guideline update from the American College of Physicians, American College of Chest Physicians, American Thoracic Society, and European Respiratory Society. Ann Intern Med. 2011; 155(3): 179–191. 10.7326/0003-4819-155-3-201108020-00008 21810710

[8] Decramer M, Rennard S, Troosters T, Mapel DW, Giardino N, Mannino D et al COPD as a Lung Disease with Systemic Consequences–Clinical Impact, Mechanisms, and Potential for Early Intervention, COPD—J Chron Obstruct Pulm Dis 2008; 5(4): 235–256. 10.1080/15412550802237531 18671149

[9] Agustí AGN. Systemic effects of Chronic Obstructive Pulmonary Disease. Proc Am Thorac Soc. 2005; 2(4): 367–370. 16267364 10.1513/pats.200504-026SR

[10] Neidell MJ. Air pollution, health, and socio-economic status: the effect of outdoor air quality on childhood asthma. J Health Econ. 2004; 23(6): 1209–1236. 15556243 10.1016/j.jhealeco.2004.05.002

[11] Siafakas NM, Vermeire P, Pride NB, Paoletti P, Gibson J, Howard P, et al Optimal assessment and management of chronic obstructive pulmonary disease (COPD): A consensus statement of the European Respiratory Society (ERS). Eur Respir J. 1995; 8(8): 398–1420. 7489808 10.1183/09031936.95.08081398

[12] Kellner P, Anderson W, Arnott D, Britton J, Jarvis M, Knapton M, et al. *Beyond Smoking Kills*: *Protecting Children*, *Reducing Inequalities* Available: http://www.ash.org.uk/beyondsmokingkills. Accessed 10 July 2015.

[13] OECD. *Health at a Glance*: *Europe 2012* Available: http://ec.europa.eu/health/reports/docs/health_glance_2012_en.pdf. Accessed 2 September 2015.

[14] Myers DG, Neuberger JS, He J. Cardiovascular effect of bans on smoking in public places. J Am Coll Cardiol 2009; 54(14): 1249–1255 10.1016/j.jacc.2009.07.022 19778665

[15] Lightwood JM, Glantz SA. Declines in acute myocardial infarction after smoke-free laws and individual risk attributable to secondhand smoke. Circulation 2009; 120: 1373–1379 10.1161/CIRCULATIONAHA.109.870691 19770392 PMC2967202

[16] Klinke ME, Jonsdottir H. Smoking addiction in chronic obstructive pulmonary disease: Integrating neurobiology and phenomenology through a review of the literature. Chron Respir Dis. 2014; 11(4): 229–236. 10.1177/1479972314546764 25150186

[17] Coogan PF, Castro-Webb N, Yu J, O'Connor GT, Palnner-JR, Rosenberg L. Active and passive smoking and the incidence of asthma in the Black Women's Health Study. Am J Respir Crit Care Med. 2015; 191(2): 168–176. 10.1164/rccm.201406-1108OC 25387276 PMC4347433

[18] Soyseth V, Johnsen HL, Kongerud J. The incidence of work-related asthma in Norwegian smelters is positively associated with dust exposure and smoking. Am J Respir Crit Care Med. 2011; 183. 10.1164/rccm.201110-1809OC 22517789

[19] Pallasaho P, Kainu A, Sovijarvi A, Lindqvist A, Piirila PL. Combined effect of smoking and occupational exposure to dusts, gases or fumes on the incidence of COPD. COPD. 2014; 11(1): 88–95. 10.3109/15412555.2013.830095 24111617

[20] Beggs PJ, Bambrick HJ. Is the global rise of asthma an early impact of anthropogenic climate change? Environ Health Perspect. 2005; 113(8): 915–919. 16079058 10.1289/ehp.7724 PMC1280328

[21] Sheffield PE, Knowlton K, Carr JL, Kinney PL. Modeling of regional climate change effects on ground-level ozone and childhood asthma. Am J Prev Med. 2011; 41(3): 251–257. 10.1016/j.amepre.2011.04.017 21855738 PMC3160600

[22] Ziska LH, Caulfield FA. Rising CO_2_ and pollen production of common ragweek (*Ambrosia artemisifolia* L.) a known allergy-inducing species: implications for public health. Aust J Plant Physiol. 2000; 27(10): 893–898.

[23] Ziska LH, Gebhard DE, Frenz DA, Faulkner S, Singer BD, Straka JG. Cities as harbingers of climate change: common ragweed, urbanization and public health. J Allergy Clin Immunol. 2003; 111(2): 290–295. 12589347 10.1067/mai.2003.53

[24] Jehn M, Donaldson G, Kiran B, Liebers U, Mueller K, Scherer D, et al Tele-monitoring reduces exacerbation of COPD in the context of climate change—a randomized controlled trial. Environ Health 2013; 12: 99: 10.1186/1476-069X-12-99 24261700 PMC3883526

[25] Vitzthum K, Scutaru C, Musial-Bright L, Quarcoo D, Welte T, Spallek M, et al Scientometric analysis and combined density-equalizing mapping of environmental tobacco smoke (ETS) research. PLOS ONE 2010; 5: e11254 10.1371/journal.pone.0011254 20582305 PMC2889821

[26] Heneberg P. Lifting the fog of scientometric research artifacts: on the scientometric analysis of environmental tobacco smoke research. J Am Soc Inf Sci Tech 2012; 64(2): 334–344

[27] Cheng K, Preston C, Ashby D, O'Hea U, Smyth RL. Time to publication as full reports of abstracts of randomized controlled trials in cystic fibrosis. Pediatr Pulmonol. 1998; 26(2): 101–105. 9727760 10.1002/(sici)1099-0496(199808)26:2<101::aid-ppul5>3.0.co;2-p

[28] de Granda-Orive JI, Rio FG, Jimenez TG, Ruiz CAJ, Reina SS, Valls RS. Analysis and evolution of bibliometric indicators of productivity and readership of articles on smoking appearing in *Archivos de Bronconeumologia* from 1970 to 2000. A comparison to other topics in respiratory medicine. Arch Bronconeumol. 2002; 38(11): 523–529. 12435318 10.1016/s0300-2896(02)75281-2

[29] Rippon I, Lewison G, Partridge MR. Research outputs in respiratory medicine. Thorax. 2005; 60(1): 63–67. 15618586 10.1136/thx.2004.031229 PMC1747152

[30] Ye B, Du TT, Xie T, Ji JT, Zheng ZH, Lioa Z, et al Scientific publications in respiratory journals from Chinese authors in various parts of North Asia: a 10-year survey of literature. BMJ Open. 2014; 4(2): e004201 10.1136/bmjopen-2013-004201 24583761 PMC3939649

[31] Frame JD, Narin F. International distribution of biomedical publications. Fed Proc., 1977; 236(6): 1790–1795 856633

[32] Lewison G, Markusova V. The evaluation of Russian cancer research. Res Eval, 2010; 19(2): 129–144.

[33] Lewison G, Sullivan R. Conflicts of interest statements on biomedical papers. Scientometrics. 2015; 102(3): 2151–2159.

[34] Sullivan R, Eckhouse S, Lewison G. *Using bibliometrics to inform cancer research policy and spending*: In: Monitoring Financial Flows for Health Research 2007: Behind the Global Numbers (Eds: Mary Ann Burke, Andrés de Francisco and Stephen Matlin), 2008; 65–78. Global Forum for Health Research, Geneva; ISBN 978-2-940401-04-8.

[35] Lewison G, Lipworth S, de Francisco A. Input indicators from output measures: a bibliometric approach to the estimation of malaria research funding. Research Evaluation, 2002; 11 (3): 155–162.

[36] Farell PM. The prevalence of cystic fibrosis in the European Union. J Cystic Fibrosis 2008; 7(5): 450–453 10.1016/j.jcf.2008.03.007 18442953

[37] Lucotte G, Hazout S, Debraekeleer M. Complete map of cystic-fibrosis mutation DF508 frequencies in Western-Europe and correlation between mutation frequencies and incidence of disease. Hum Biol. 1995; 67(5): 797–803. 8543293

[38] Public Health England. *Respiratory Profiles for North East England* Available: http://www.nepho.org.uk/respiratory/. Accessed 2 September 2015.

[39] Dodge JA, Lewis PA, Stanton M, Wilsher J. Cystic fibrosis mortality and survival in the UK: 1947–2003. Eur Respir J. 2007; 29(3): 522–526. 17182652 10.1183/09031936.00099506

